# Serelaxin Protects H9c2 Cardiac Myoblasts against Hypoxia and Reoxygenation-Induced Damage through Activation of AMP Kinase/Sirtuin1: Further Insight into the Molecular Mechanisms of the Cardioprotection of This Hormone

**DOI:** 10.3390/antiox13020163

**Published:** 2024-01-27

**Authors:** Virginia Zizi, Matteo Becatti, Daniele Bani, Silvia Nistri

**Affiliations:** 1 Department of Experimental & Clinical Medicine, Research Unit of Histology & Embryology, University of Florence, Viale G. Pieraccini 6, 50139 Florence, Italy; virginia.zizi@unifi.it (V.Z.); daniele.bani@unifi.it (D.B.); 2 Department of Experimental & Clinical Biomedical Sciences “Mario Serio”, Section of Biochemical Sciences, University of Florence, Viale G.B. Morgagni 50, 50134 Florence, Italy; matteo.becatti@unifi.it

**Keywords:** Serelaxin, SIRT1, AMPK, hypoxia–reoxygenation, apoptosis, oxidative stress, H9c2 cells

## Abstract

Serelaxin (RLX), namely the human recombinant Relaxin-2 hormone, protects the heart from ischemia/reperfusion (I/R)-induced damage due to its anti-inflammatory, anti-apoptotic and antioxidant properties. RLX acts by binding to its specific RXFP1 receptor whereby it regulates multiple transduction pathways. In this in vitro study, we offer the first evidence for the involvement of the AMP kinase/Sirtuin1 (AMPK/SIRT1) pathway in the protection by RLX against hypoxia/reoxygenation (H/R)-induced damage in H9c2 cells. The treatment of the H/R-exposed cells with RLX (17 nmol L^−1^) enhanced SIRT1 expression and activity. The inhibition of SIRT1 signaling with EX527 (10 µmol L^−1^) reduced the beneficial effect of the hormone on mitochondrial efficiency and cell apoptosis. Moreover, RLX upregulated the AMPK pathway, as shown by the increase in the expression of phospho-AMPK-activated protein. Finally, AMPK pathway inhibition by Compound C (10 and 20 μmol L^−1^) abrogated the increase in SIRT1 expression induced by RLX, thus suggesting the involvement of the AMPK pathway in this effect of RLX. These results strengthen the concept that RLX exerts its cardioprotective effects against H/R-induced injury through multiple pathways which also include AMPK/SIRT1. These new findings support the use of RLX or RLX-derived molecules as a promising therapeutic for those diseases in which I/R and oxidative stress play a pathogenic role.

## 1. Introduction

Serelaxin (RLX), the recombinant form of human Relaxin-2, is a natural peptide hormone originally known for its effects on the female reproductive system, which primarily include lengthening of the pubic symphysis and softening of the tissues of the birth canal, stimulation of the mammary gland and endometrial development, and the maintenance of myometrial quiescence [1]. RLX has long been recognized as a pleiotropic hormone, being able to mediate a broad range of actions across a variety of tissue and organs in both sexes, including the cardiovascular system [2,3]. In fact, RLX is produced by cardiomyocytes [4], and RLX receptors have been chiefly detected in the atrial myocardium [5]. Evidence from preclinical studies indicated that RLX exerts chronotropic, inotropic and anti-arrhythmic action on the myocardium and it is able to increase cardiac perfusion both by dilating the pre-existing coronary circulation and by promoting neo-angiogenesis [6]. Experimental evidence in vivo and in vitro, from independent research groups worldwide, attests that RLX is able to protect the heart from the damage induced by ischemia and reperfusion (I/R) [7,8,9,10,11,12]. In these models, RLX markedly reduces several parameters of myocardial injury and the extension of nonviable myocardium, and improves cardiac contractile performance [7,8,9,10,11,12]. The protective action of RLX on myocardial injury is attributable not only to its anti-inflammatory, anti-apoptotic and antifibrotic properties, but also to its ability to reduce oxidative stress [12,13]. In fact, in H9c2 cardiac muscle cells exposed to hypoxia and reoxygenation (H/R), RLX reduces the production of reactive oxygen species (ROS), improves mitochondrial activity impaired by H/R and prevents related cell apoptosis [12]. RLX, although it does not possess intrinsic antioxidant properties, is capable of potentiating cardiomyocyte resistance to oxygen deprivation and nitroxidative stress through a mechanism that allows it to increase the cellular availability of reduced glutathione (GSH), a ubiquitous endogenous antioxidant metabolite [12].

RLX exerts its multiple actions on the heart by binding to a specific G protein-coupled receptor, referred to as RLX family peptide receptor 1 (RXFP1), which is bioactive in rodent animal models and cells [13,14]. When RLX binds to the RXFP1 receptor, it activates multiple signal transduction pathways, including the cyclic nucleotide systems (i.e., adenylate cyclase/cAMP, nitric oxide (NO)/guanylate cyclase/cGMP), phosphatidylinositol-4,5-bisphosphate 3-kinase (PI3K)/Akt, Extracellular signal-regulated kinase-1/2 (ERK1/2) [15,16] and AMP-activated protein kinase (AMPK) [17], depending on the cell type studied and on the differences in G protein coupling. Moreover, recent studies have demonstrated that some physiological actions of RLX are mediated by crosstalk with signaling cascades activated by other receptors, including TGF-β receptors, angiotensin II type 2 receptor and Notch1 [8,16]. The ability of RLX/RXFP1 to upregulate multiple cellular transduction signaling could explain the pleiotropic effect of RLX on cardioprotection.

Silent information regulator 1 (SIRT1) is a nicotinamide adenine dinucleotide (NAD)-dependent histone deacetylase belonging to the highly conserved Sirtuin family which, in mammals, consists of seven members (SIRT1–SIRT7), with different subcellular localization, enzymatic activity and binding targets [18,19,20]. SIRT1 is ubiquitously expressed and, through the deacetylation of a variety of histones and non-histone proteins, regulates multiple biological processes, such as cellular senescence, metabolism, energy, redox balance and apoptosis [19,20]. SIRT1 is also an essential regulator of myocardial I/R-induced injury [21]. Numerous studies demonstrate that SIRT1 protects the heart from I/R-induced damage via multiple cellular mechanisms, which include a reduction in inflammation and apoptosis, the preservation of mitochondrial function and the attenuation of oxidative stress. SIRT1 exerts its antioxidant action by stimulating the upregulation of antioxidant enzymes and decreasing ROS production [21,22,23,24]. SIRT1 activity is enhanced by AMPK, a serine/threonine protein kinase complex which plays a crucial role in cell energy, metabolism and survival [19,25]. The stimulation of the AMPK pathway by different stimuli results in an increase in cellular NAD+ levels, which, in turn, sustain the SIRT1-mediated deacetylation of downstream protein targets, including antioxidant enzymes [24].

The aim of this in vitro study is to investigate the possible involvement of the AMPK/SIRT1 pathway in the noted protective action of RLX on H/R-induced damage.

## 2. Materials and Methods

### 2.1. Cell Cultures and Treatments

H9c2 embryonic rat cardiac muscle cells, a well-characterized and widely used cell line to study myocardial cell ischemia [26], were obtained from the European Collection of Cell Cultures (ECACC, Salisbury, UK). The cells were cultured in Dulbecco’s modified Eagle’s medium (DMEM) supplemented with 10% heat-inactivated fetal bovine serum (Sigma-Aldrich, Milan, Italy), 2 mmol L^−1^ glutamine, 250 U mL^−1^ penicillin G and 250 μg mL^−1^ streptomycin (Sigma-Aldrich), in a humidified atmosphere with 5% CO_2_ at 37 °C.

H9c2 cells were subjected to H/R, simulated in vitro by substrate starvation plus hypoxia followed by reoxygenation, as previously described [16]. H9c2 cells were incubated in DMEM with no serum or glucose, placed in a hypoxic modular incubator chamber (Billups-Rothenberg, Inc., San Diego, CA, USA), gassed with 95% N_2_ and 5% CO_2_, humidified and warmed at 37 °C for 5 h. A flow meter was used to measure the quantity of gas mixture introduced into the chamber (25 L min^−1^). At the end of hypoxia, the medium was replaced with fresh complete culture medium and the cells were reoxygenated for 2 h through incubation in normoxic conditions. Control normoxic cultures were also prepared.

Cells were treated with human recombinant RLX, kindly provided by the RRCA Relaxin Foundation (Florence, Italy), at concentrations of 17 nmol L^−1^, or left untreated. RLX was added at the onset of hypoxia and maintained throughout the experimental period until the end of reoxygenation. This RLX concentration was similar to that used in the previous experiments to demonstrate its ability to protect H9c2 cells from H/R-induced injury [8]. The experiments were performed at least in triplicate.

To assess the involvement of the SIRT1 pathway in the protective action of RLX against H/R-induced cell damage, H9c2 cells were treated with the specific SIRT1 inhibitor EX527 (1 and 10 μmol L^−1^) (Sigma Aldrich) 2 h before the onset of hypoxia. The chosen concentrations and exposure time of the EX527 inhibitor were as reported in [27,28].

To investigate the involvement of AMPK signaling in the possible stimulation of SIRT1 by RLX, H9c2 cells were treated for 24 h before the onset of hypoxia with 10 and 20 μmol L^−1^ of Compound C (Dorsomorphin) (MedChemExpress, Monmouth Junction, NJ, USA), a selective and ATP-competitive AMPK inhibitor. The chosen concentrations and exposure time of Compound C were as reported in [29].

### 2.2. Western Blotting

H9c2 cells from the different experimental groups were lysed in cold buffer (Cell Signaling, Danvers, MA, USA) composed of 20 mmol L^−1^ Tris/HCl pH 7.5, 150 mmol L^−1^ NaCl, 1 mmol L^−1^ Na_2_EDTA, 1 mmol L^−1^ EGTA, 1% Triton X-100, 2.5 mmol L^−1^ sodium pyrophosphate, 1 mmol L^−1^ β-glycerophosphate, 1 mmol L^−1^ Na_3_VO_4_ and 1 mg mL^−1^ leupeptin, to which we added 1 mmol L^−1^ phenylmethylsulfonyl fluoride (PMSF) protease inhibitor (Sigma Aldrich). Total protein content was measured spectrophotometrically using a micro-BCA^TM^ Protein Assay Kit (ThermoFisher Scientific, Waltham, MA, USA). Forty µg of total proteins were electrophoresed by SDS–PAGE and blotted onto polyvinylidene difluoride (PVDF) membranes (Millipore, Bedford, MA, USA). The membranes were rinsed with T-PBS (20 mmol L^−1^ Tris-HCl buffer, 150 mmol L^−1^ NaCl and 0.1% Tween 20) and incubated with 5% bovine serum albumin (BSA) (Sigma Aldrich) or non-fat dry milk (Sigma Aldrich) diluted in T-PBS for 2 h at room temperature, under stirring, to prevent non-specific peptide binding. Then, the membranes were incubated overnight at 4 °C, under stirring, with the following primary antibodies: rabbit polyclonal anti-SIRT1 (1:800; Santa Cruz Biotechnology, Dallas, TX, USA), rabbit polyclonal anti-AMPKα (1:1000; Cell Signaling), rabbit polyclonal anti-phospho-AMPKα (1:1000; Cell Signaling), rabbit polyclonal anti-caspase 3 (1:1000; Cell Signaling), which detects both the full length and the cleaved form of the protein, and mouse monoclonal anti-β-actin (1:4500; Sigma Aldrich), assuming β-actin as control invariant protein. After three rinses with T-PBS, membranes were incubated with rabbit or mouse peroxidase-labeled secondary antibodies (1:15,000; Vector Laboratories, Burlingame, CA, USA) for 1 h at room temperature, under stirring. All the antibodies used were diluted in T-PBS containing 1% BSA. Specific bands were detected using enhanced chemiluminescent substrate (Luminata Crescendo Western HRP substrate, Merck Millipore, Darmstadt, Germany). Densitometric analysis of the bands was performed using Scion Image Beta 4.0.2 image analysis software (Scion Corp., Frederick, MD, USA) and the values were normalized to β-actin.

### 2.3. SIRT1 Activity

SIRT1 activity was measured using an SIRT1 Activity Fluorometric Assay Kit (Abcam, Cambridge, UK), according to the manufacturer’s protocol. Briefly, H9c2 cells from each experimental group were lysed with an appropriate buffer that did not contain protease inhibitors to avoid their interference with the development of the assay. The lysis buffer was composed of 20 mmol L^−1^ Tris-HCl (pH 7.5), 250 mmol L^−1^ NaCl, 1 mmol L^−1^ EDTA, 1 mmol L^−1^ EGTA, 1% Triton X-100 and 1 mmol L^−1^ dithiothreitol (DTT). Total protein content was measured spectrophotometrically using a micro-BCA^TM^ Protein Assay Kit (ThermoFisher Scientific). Four hundred micrograms of total protein extracts were immunoprecipitated for SIRT1. To preclean and minimize extra bands resulting from nonspecific precipitation, cell lysates were incubated with Protein G PLUS/Protein A-Agarose suspension (Calbiochem, Darmstadt, Germany) for 1 h and 30 min, at 4 °C, under stirring. After centrifugation, the samples were incubated with rabbit polyclonal anti-SIRT1 (1:100; Santa Cruz Biotechnology) overnight, at 4 °C, under stirring. Then, Protein G PLUS/Protein A-Agarose suspension was added to each sample and incubated for 2 h, at 4 °C, under stirring. After centrifugation, the obtained precipitates were used to measure SIRT1 activity. Fluorescence intensity was read for 30 to 60 min at 1- to 2-min intervals using the microtiter plate fluorometer “Infinite M200 PRO” (Tecan, Männedorf, Switzerland) with excitation at 340–360 nm and emission at a 440–460 nm wavelength. The rate of reaction was measured and calculated while the reaction velocity remained constant.

### 2.4. Mitochondrial Activity

Cell mitochondrial activity was assayed by the 3-(4,5-dimethylthiazol-2-yl)-2,5-diphenyl tetrazolium bromide (MTT) assay (Sigma Aldrich), a tetrazolium salt, which is rapidly reduced to insoluble formazan by the cells’ mitochondrial enzymes. Therefore, this assay is commonly used to determine both mitochondrial activity and, indirectly, cell survival. Briefly, H9c2 cells (5 × 10^4^/well) were seeded in 24-well plates and subjected to H/R in the presence or absence of RLX and of the SIRT1 inhibitor EX527 (1 and 10 μmol L^−1^). At the end of the treatments, the culture medium was removed from each well and replaced with 300 μL of MTT (0.5 mg mL^−1^), followed by incubation for 4 h at 37 °C. Then, 350 μL of dimethyl sulfoxide (DMSO) (Sigma Aldrich) was added to each well to dissolve the formazan crystals. The plate was gently shaken for 5 min at room temperature and read at a 550 nm wavelength on an “Infinite M200 PRO” plate reader (Tecan). Optical density was assumed to be an indicator of mitochondrial activity.

### 2.5. Statistical Analysis

The reported data are expressed as the mean ± SEM of no less than 3 independent experiments. A Shapiro–Wilk normality test for small size samples was first used to assess the gaussian distribution of the experimental data. A Statistical comparison of differences between groups was carried out using one-way ANOVA followed by a Tukey multiple comparison test for the parametric values or a Kruskal–Wallis test followed by Dunn’s multiple comparison test for the non-parametric values. To compare the average values of two datasets, Student’s unpaired *t*-test for parametric values or a Mann–Whitney test for nonparametric values was performed. A *p* value ≤ 0.05 was considered significant. Calculations were carried out using the GraphPad Prism 10.0 statistical program (GraphPad Software, San Diego, CA, USA).

## 3. Results

### 3.1. RLX Activates SIRT1 Signaling in H9c2 Cells Subjected to H/R

**Figure 1:**
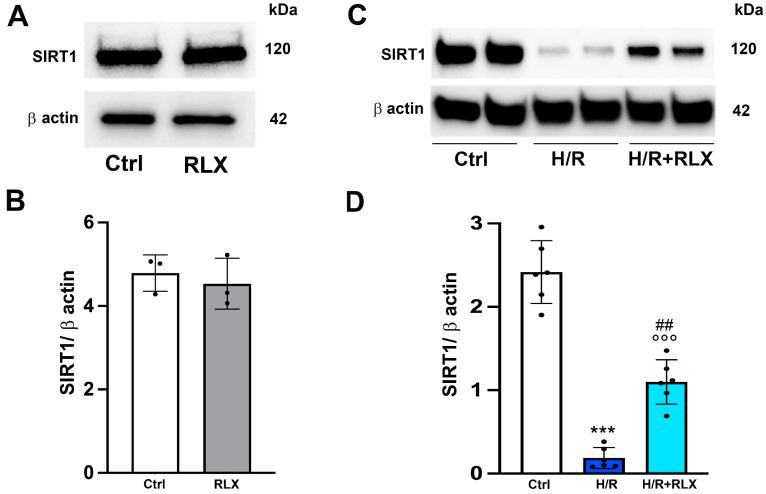
RLX counteracts the decrease in SIRT1 expression in H9c2 cells exposed to H/R. Representative images of Western blotting and quantitative analysis showing SIRT1 and β-actin expression in H9c2 cells under normoxia (n = 3) (**A**,**B**) and upon H/R challenge (n = 6) (**C**,**D**). Significance of differences (**B**: Mann–Whitney nonparametric Test; **D**: one-way ANOVA and Tukey multiple comparison test): *** *p* < 0.001 vs. controls (Ctrl); °°° *p* < 0.001 vs. controls (Ctrl); ## *p* < 0.01 vs. H/R.

**Figure 2:**
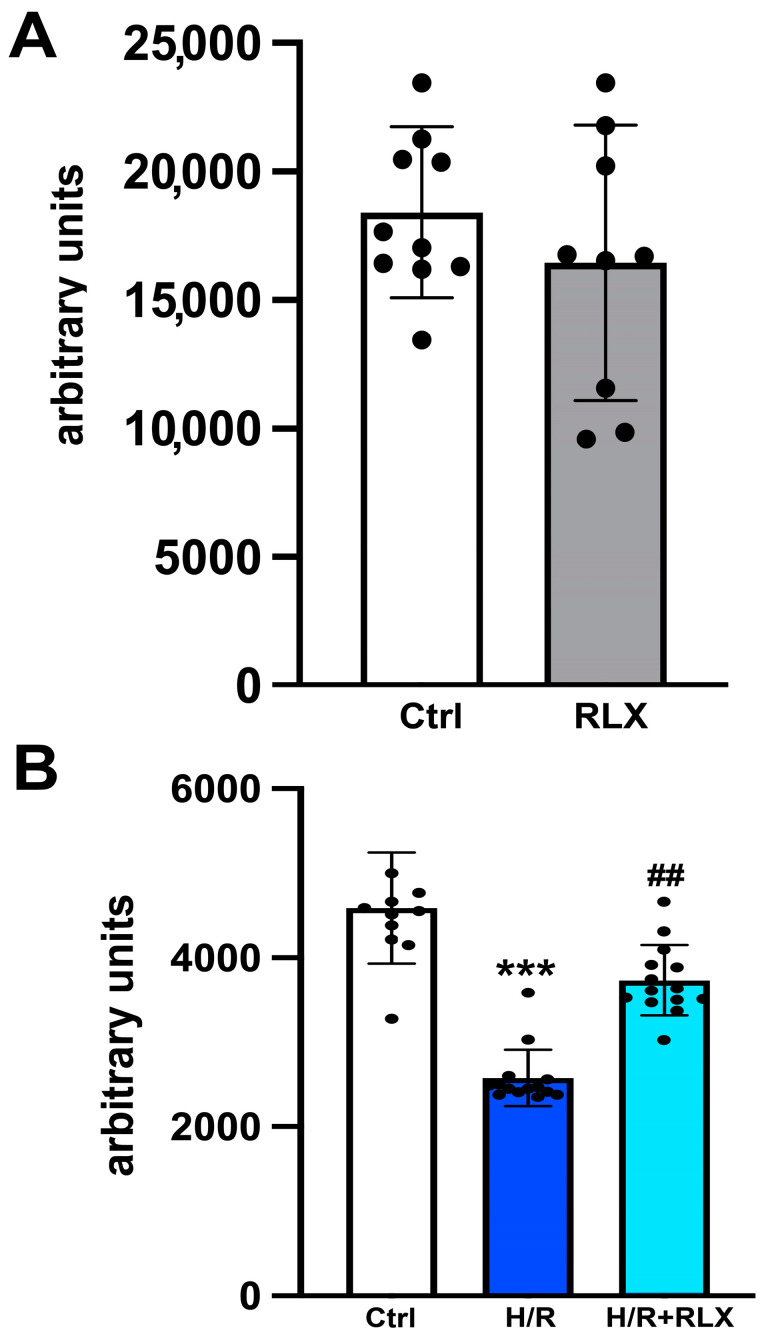
RLX counteracts the decrease in SIRT1 activity in H9c2 cells exposed to H/R. Representative diagrams showing SIRT1 activity in H9c2 in normoxia (n = 9–10) (**A**) and upon H/R challenge (n = 10–14) (**B**). Significance of differences (**A**: Student’s unpaired *t*-test; **B**: Kruskal–Wallis test and Dunn multiple comparison test): *** *p* < 0.001 vs. controls (ctrl); ## *p* < 0.01 vs. H/R.

We investigate the involvement of SIRT1 signaling in the mechanism of action by which RLX protects cells from H/R-induced damage. The treatment of the control cells not subjected to H/R with RLX (17 nmol L^−1^) had no effect on SIRT1 expression (Figure 1A,B) or activity (Figure 2A). In H9c2 cells subjected to H/R, SIRT1 expression and total activity were significantly reduced compared with the control cells (Figure 1C,D and Figure 2B). Treatment of the H/R-exposed cells with RLX (17 nmol L^−1^) significantly increased both SIRT1 expression (Figure 1C,D) and total activity (Figure 2B).

### 3.2. RLX Induces Cell Protection through Stimulation of SIRT1 Signaling

**Figure 3:**
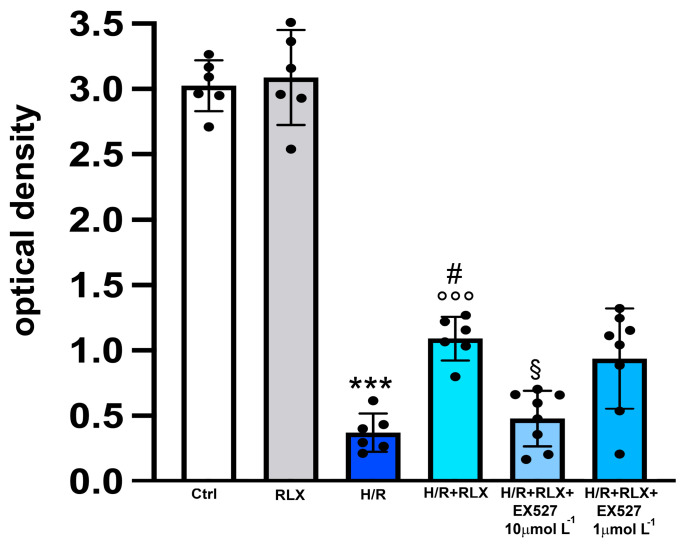
Inhibition of SIRT1 signaling counteracts the beneficial effects of RLX on mitochondrial respiratory chain efficiency in H9c2 cells exposed to H/R. Representative diagrams showing mitochondrial activity, assayed by the MTT assay, of H9c2 cells in normoxia (Ctrl) (n = 6) and under H/R in the absence (n = 6) and in the presence of RLX (n = 6) and upon treatment with the selective SIRT1 inhibitor, EX527 1 and 10 µmol L^−1^ (n = 6–8). Significance of differences (one-way ANOVA and Tukey multiple comparison test): *** *p* < 0.001 vs. controls (Ctrl); # *p* < 0.05 vs. H/R; °°° *p* < 0.001 vs. controls (Ctrl) and RLX alone; § *p* < 0.05 vs. H/R+RLX.

In H9c2 cells subjected to H/R, the efficiency of the mitochondrial respiratory chain, measured by the MTT assay, was significantly reduced compared with the control cells (Figure 3). Treatment with RLX (17 nmol L^−1^) significantly improved mitochondrial activity impaired by H/R. This beneficial effect of RLX was significantly reduced, albeit not abolished, in the presence of the SIRT1 inhibitor EX527 at the higher concentration (10 µmol L^−1^) (Figure 3). Treatment of the control H9c2 cells with RLX (17 nmol L^−1^) did not influence basal mitochondrial efficiency (Figure 3). The same data can also be assumed to be indirect parameters of cell viability; in this way, the percentage reductions with respect to the controls, assumed to be 100%, were H/R: 12.7; H/R+RLX: 36; H/R+RLX+EX527 1 µmol L^−1^: 31; and H/R+RLX+EX527 10 µmol L^−1^: 15.8.

**Figure 4:**
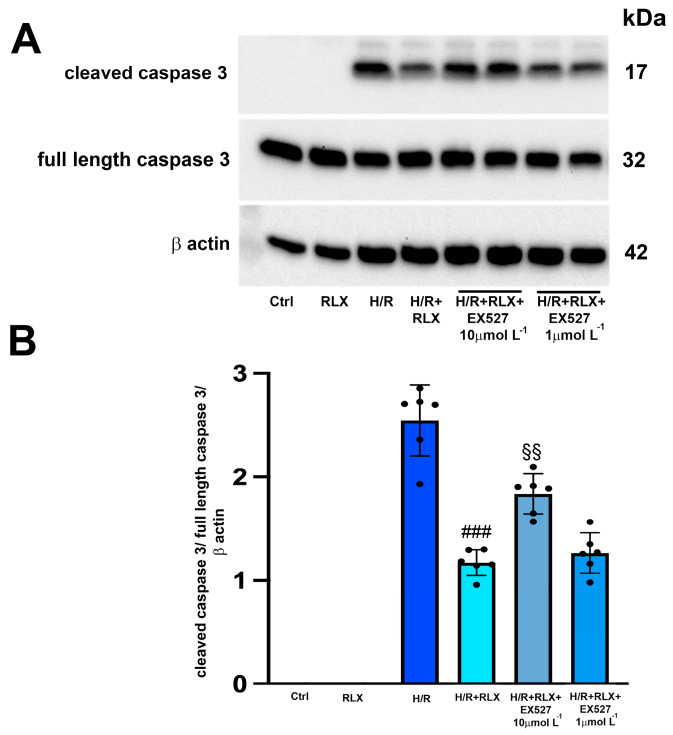
Inhibition of SIRT1 signaling counteracts the beneficial effects of RLX on apoptosis of H9c2 cells exposed to H/R. Representative image of Western blotting (**A**) and quantitative analysis (**B**) showing the expression of cleaved and full-length caspase 3 and β-actin in H9c2 cells under normoxia (Ctrl, n = 3–4) and upon H/R challenge in the presence or absence of the selective SIRT1 inhibitor EX527 (1 and 10 µmol L^−1^, n = 6). Significance of differences (one-way ANOVA and Tukey multiple comparison test): ### *p* < 0.001 vs. H/R; §§ *p* < 0.01 vs. H/R+RLX.

The H/R challenge induced the expression of the cleaved active caspase 3, leaving the expression of the full-length caspase 3 unaltered (Figure 4A,B). As expected, treatment with RLX (17 nmol L^−1^) significantly reduced the expression of cleaved caspase 3 compared with the H/R-induced cells (Figure 4A,B). The protective effect of RLX on apoptosis was significantly reduced, but not completely abolished, by co-treatment with the SIRT1 inhibitor EX527 at the higher concentration (10 µmol L^−1^), as judged by the observed increase in cleaved caspase 3 expression compared with the RLX-treated cells (Figure 4A,B). Full-length caspase 3 expression was not affected by the different treatments (Figure 4A,B). In H9c2 cells not subjected to H/R, treated or not treated with RLX (17 nmol L^−1^), the active, cleaved form of caspase 3 was not detected (Figure 4A,B).

### 3.3. RLX Affects SIRT1 Signaling through Activation of AMPK Pathway

**Figure 5:**
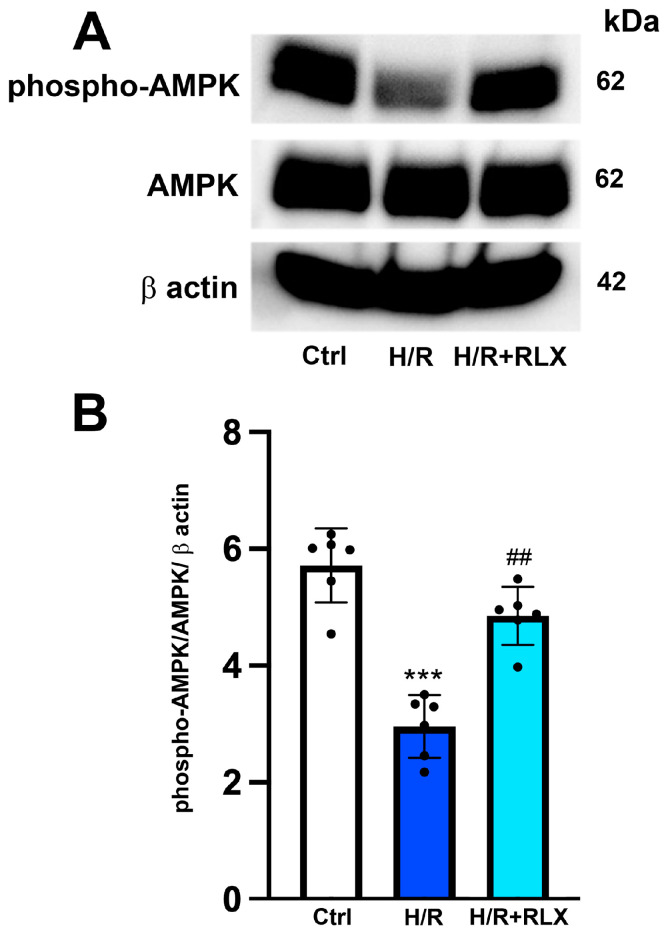
RLX treatment activates AMPK signaling in H9c2 cells exposed to H/R. Representative images of Western blotting (**A**) and quantitative analysis (**B**) showing phospho-AMPK, AMPK and β-actin expression in H9c2 cells upon H/R challenge (n = 6). Significance of differences (one-way ANOVA and Tukey multiple comparison test): *** *p* < 0.001 vs. controls (Ctrl); ## *p* < 0.01 vs. H/R.

The possible molecular mechanism linking the activation of the RXFP1 receptor by RLX and the stimulation of SIRT1 signaling was also investigated, focusing on the involvement of the AMPK pathway. In H9c2 cells exposed to H/R, phospho-AMPKα expression, the active form of the protein, is significantly decreased compared with the control cells (Figure 5A,B). Treatment with RLX (17 nmol L^−1^) induced a significant increase in phospho-AMPKα expression compared with HR-exposed cells, leaving total AMPKα expression unchanged (Figure 5A,B).

**Figure 6:**
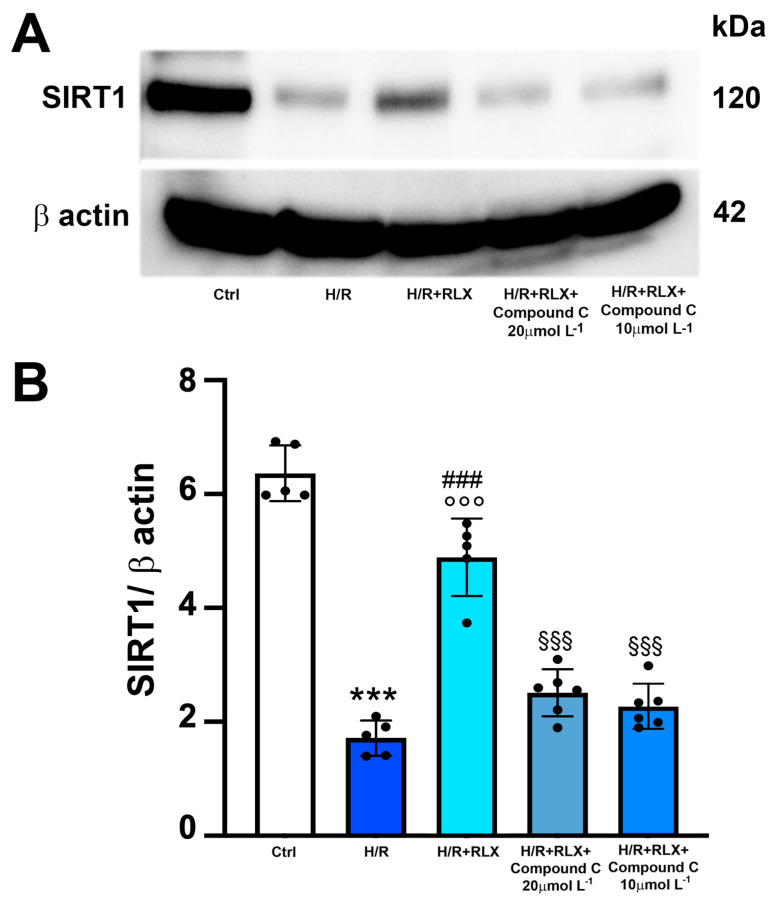
Inhibition of AMPK signaling counteracts the increase in SIRT1 expression induced by RLX in H9c2 cells exposed to H/R. Representative images of Western blotting (**A**) and quantitative analysis (**B**) showing SIRT1 and β-actin expression in H9c2 cells upon H/R challenge in the presence or absence of the selective AMPK inhibitor Compound C (10 and 20 µmol L^−1^) (n = 5–6). Significance of differences (one-way ANOVA and Tukey multiple comparison test): *** *p* < 0.001 vs. controls (Ctrl); ### *p* < 0.001 vs. H/R; °°° *p* < 0.001 vs. controls (Ctrl); §§§ *p* < 0.001 vs. H/R+RLX.

Then, to assess whether the activation of the AMPK pathway by RLX underlies the ability of the hormone to activate SIRT1 signaling, we evaluated SIRT1 signaling in the presence of the selective AMPK inhibitor Compound C. The co-incubation of H/R-exposed H9c2 cells with RLX and Compound C (10 and 20 µmol L^−1^) significantly abated the activation of SIRT1 signaling induced by RLX (Figure 6A,B).

## 4. Discussion

In recent years, robust evidence has been accumulating that the peptide hormone RLX can markedly reduce tissue and cell damage in pathological events that involve oxidative stress, such as vascular and lung injury caused by exposure to cigarette smoke [30] and myocardial damage induced by I/R [3,7,8]. RLX exerts its protective effects on oxidative stress by binding to its cognate G protein-coupled receptor RXFP1 and activating multiple intracellular transduction pathways through which it can regulate the expression and/or the activity of different cell protein kinases, including PI3K/AKT, ERK1/2 and AMPK [13,14,17]. This in vitro study provides the first evidence that RLX is able to activate SIRT1 signaling in H/R-exposed H9c2 cells through the stimulation of the AMPK pathway. The treatment of H9c2 cells with RLX, from the onset of hypoxia to the end of reoxygenation, increases SIRT1 expression, resulting in an increase in total SIRT1 activity, which is reduced by H/R. Through this mechanism, RLX protects these cells against H/R-induced oxidative damage. In fact, SIRT1 signaling inhibition by EX527 reduces, albeit not abolishes, the beneficial effects of the hormone on mitochondrial efficiency and cell apoptosis. The finding that SIRT1 inhibition does not completely abolish the effects of RLX confirms that multiple cytoprotective signaling pathways are operated by this hormone in cardiac muscle cells [8,12,15]. Of note, RLX is able to increase SIRT1 signaling only under pathological conditions, such as H/R, as it has no effects on SIRT1 expression and activity in the control cells under basal conditions. Within the limitation of this cellular model, SIRT1 seems to only be activated by RLX under stress conditions as a defensive mechanism. This agrees with the well-known cardioprotective properties of SIRT1. Numerous studies have shown that SIRT1 signaling plays a fundamental role in protecting the heart from I/R-induced injury [21,31] since its upregulation inhibits inflammation, apoptosis, fibrosis and oxidative stress [32,33], whereas its downregulation or inactivation predisposes the myocardium to oxidative damage. In turn, oxidative stress causes the inactivation of SIRT1 [34], thus sparkling a vicious cycle which leads to the exacerbation of heart injury [19,35]. However, further studies need to be conducted to better understand the exact role of the SIRT1 pathway in the cardioprotective action of RLX.

In addition, this study provides evidence that RLX stimulates SIRT1 signaling via the activation of AMPK, a serine/threonine protein kinase complex whose upregulation reduces the inflammatory response and oxidative stress [36,37,38]. The SIRT1 and AMPK pathways have a close interaction in the regulation of cellular energy, metabolism, aging and the response to oxidative stress, since they can reciprocally enhance each other’s activity [39,40,41]. In fact, AMPK enhances SIRT1 activity by increasing the cellular levels of the SIRT1 cofactor NAD+ [25], and, in turn, SIRT1 stimulates the AMPK pathway via the deacetylation and activation of specific upstream regulators of AMPK signaling [42]. Moreover, the activation of AMPK/SIRT1 signaling results in the downregulation of pro-inflammatory cytokines, a reduction in ROS production and the upregulation of antioxidant enzymes [43]. In this study, we have demonstrated that RLX reverts the reduction in phospho-AMPK expression induced by H/R while leaving total AMPK unchanged. This suggests a direct regulatory action of the hormone on the AMPK protein without affecting its gene expression. It is conceivable that SIRT1 activation may quickly occur after RLX binding to RXFP1, thus contributing to explaining why the previously reported protective effects of RLX on myocardial H/R occurred in the short term, soon after RLX administration at reperfusion [7,8]. Moreover, the treatment of H/R-induced H9c2 cells with RLX in the presence of the selective AMPK inhibitor Compound C abolished the ability of the hormone to activate the SIRT1 pathway, thus confirming the involvement of AMPK on SIRT1 activation by RLX. The activation by RLX of the AMPK/SIRT1 pathway could explain the possible mechanism by which RLX increases GSH bioavailability in H9c2 cells challenged with H/R [9]. It is well known that SIRT1 upregulates the expression and activity of nuclear factor erythroid 2-related factor 2 (Nrf2) [44], a transcription factor involved in the expression of glutathione reductase and de novo synthesis of GSH [45]. This hypothesis is worthy of being explored in future investigations.

We point out that the present data were obtained from cells in culture; this represents a substantial limitation of this study because the reported findings cannot be directly translated to an in vivo condition, not to mention a clinical setting.

## 5. Conclusions

The results of the present study expand the current knowledge on the molecular signal transduction mechanisms operated by RLX and its cognate receptor RXFP1 in cardiac H9c2 cells. The data obtained offer evidence for the involvement of the SIRT1 pathway in the protective action of this hormone against cell oxidative damage induced by H/R and strengthen the concept that RLX exerts its protective effects via multiple signaling pathways. From a clinical perspective, these results corroborate the current experimental background supporting RLX or RLX-derived molecules as promising new therapeutics for those diseases in which I/R and oxidative stress play a pathogenic role. This view is further supported by the notion that RLX stimulates the NO pathway [16,46], which, together with carbon monoxide (CO), is regarded as one of the youngest gaseous mediators holding promise in pharmacotherapy [47,48].

## Data Availability

The datasets generated and/or analyzed during the current study are available from the corresponding author upon reasonable request.
